# Melatonin, Noncoding RNAs, Messenger RNA Stability and Epigenetics—Evidence, Hints, Gaps and Perspectives

**DOI:** 10.3390/ijms151018221

**Published:** 2014-10-10

**Authors:** Rüdiger Hardeland

**Affiliations:** Johann Friedrich Blumenbach Institute of Zoology and Anthropology, University of Göttingen, Berliner Str. 28, Göttingen D-37073, Germany; E-Mail: rhardel@gwdg.de; Tel.: +49-551-395414

**Keywords:** circadian, DNA methylation, histone acetylation, histone methylation, lncRNA, melatonin, miRNA, piRNA, RNA deadenylases

## Abstract

Melatonin is a highly pleiotropic regulator molecule, which influences numerous functions in almost every organ and, thus, up- or down-regulates many genes, frequently in a circadian manner. Our understanding of the mechanisms controlling gene expression is actually now expanding to a previously unforeseen extent. In addition to classic actions of transcription factors, gene expression is induced, suppressed or modulated by a number of RNAs and proteins, such as miRNAs, lncRNAs, piRNAs, antisense transcripts, deadenylases, DNA methyltransferases, histone methylation complexes, histone demethylases, histone acetyltransferases and histone deacetylases. Direct or indirect evidence for involvement of melatonin in this network of players has originated in different fields, including studies on central and peripheral circadian oscillators, shift work, cancer, inflammation, oxidative stress, aging, energy expenditure/obesity, diabetes type 2, neuropsychiatric disorders, and neurogenesis. Some of the novel modulators have also been shown to participate in the control of melatonin biosynthesis and melatonin receptor expression. Future work will need to augment the body of evidence on direct epigenetic actions of melatonin and to systematically investigate its role within the network of oscillating epigenetic factors. Moreover, it will be necessary to discriminate between effects observed under conditions of well-operating and deregulated circadian clocks, and to explore the possibilities of correcting epigenetic malprogramming by melatonin.

## 1. Introduction

Melatonin is a highly pleiotropic regulator molecule that influences countless functions in numerous organs and cell types [1]. This includes the modulation of gene expression in a number of documented cases [2,3,4,5,6]. Over the course of recent years, control mechanisms of gene expression have turned out to be much more complex than previously believed. Our previous concepts were mainly focused on activating or repressing transcription factors as well as on interactions with nucleosomes to be removed in the case of gene activation. This view has meanwhile been shown to be entirely insufficient. Even the earlier discoveries of posttranscriptional regulation mechanisms were far from a presentiment of the actually known complexity of the processes by which formation and translation of mRNAs can be enhanced or shut off.

The multitude of control levels comprises epigenetic mechanisms in the classic sense, such as DNA methylation in promoters and other sites important for, e.g., utilisation of exons or regulatory sections of introns wherever CpG islands are found [7,8,9,10], chromatin remodeling via histone modification [11,12,13,14], and the previously enigmatic imprinting of alleles [15]. Moreover, an unforeseeably high number of noncoding RNAs (ncRNAs) has been discovered, whose importance is increasingly perceived. Countless microRNAs (miRNAs) have been described in recent years [16,17], and their actions exceed the first-discovered dicer- and argonaute-dependent processes observed in small interfering RNA-(siRNA)-associated knockdowns [16,17,18,19]. Other small RNA species have been additionally identified, such as piRNAs (PIWI-interacting RNAs) [18,20], snoRNAs (small nucleolar RNAs) [15,21], circRNAs (circular RNAs) [17], and telomeric RNAs [22]. An additional surprise resulted from the discovery of long noncoding RNAs (lncRNAs) [23,24,25], a group of functionally divergent molecules that can act as scaffolds for regulatory protein complexes, influence heterochromatin stability, transcription, splicing and translation, or can themselves be spliced into siRNAs, snoRNAs and residual RNAs with other roles. A particularly remarkable fact is the quantitative extent of ncRNA formation. The transcriptome exceeds by far the total amount of coding genes, and this is even valid for the quantity of lncRNAs relative to mRNAs [23]. The lncRNAs can originate from intergenic regions (lincRNAs) [26], overlap with genes, or even represent antisense transcripts [23,24,27,28,29]. The usage and stability of mRNAs does not only depend on ncRNAs, especially, miRNAs, but is also influenced by deadenylases that shorten poly(A) tails [30,31,32]. Sometimes, deadenylases have been shown to exert additional effects, such as stabilization of other mRNA species, as reported for nocturnin (NOC) [33].

In the light of the above, any up- or down-regulation of gene expression by melatonin should be assumed to be potentially modulated by ncRNAs and other epigenetic mechanisms. Moreover, melatonin biosynthesis, in the pineal gland or in extrapineal organs, is based on gene expression and, therefore, influenced by some of the mechanisms summarized. Epigenetic effects that modulate the actions of melatonin were first assumed to exist in 1994, in a gerontological context [34], however, in the absence of direct evidence. This topic was re-addressed in more recent publications, with regard to NF-κB and Nrf2 signaling [5,35,36]. Meanwhile, additional information has accumulated for connections between melatonin and epigenetic mechanisms. However, this is still a field in its infancy and the findings are far from showing a coherent picture. On the other hand, there are numerous areas in which effects of melatonin are well documented and in which epigenetic changes by other factors have been shown. Such findings may indicate epigenetic nexus to melatonin. Pertinent data have been obtained in fields as different as inflammation, oxidative stress, cancer, shift work, aging, energy expenditure/obesity, diabetes type 2, neuropsychiatric disorders, and neurogenesis. Moreover, a particularly important body of evidence has emerged from studies on circadian rhythms, in which most of the above-mentioned epigenetic mechanisms have been shown to be involved. With regard to the synchronizing and orchestrating role of melatonin for both central and peripheral circadian oscillators [37,38], interactions between identified epigenetic players within the circadian system and melatonin are highly likely. It is an aim of this review to direct readers to these possible connections and to the presumably exceptional importance of epigenetic research in future studies on melatonin.

## 2. Melatonin Synthesizing Organs: Modulation of mRNA Stability and Demonstration of Cycling lncRNAs

With regard to the notion that ncRNAs and deadenylases contribute to the levels of gene expression in, perhaps, every nucleate mammalian cell, it is not surprising that such molecules also modulate the biosynthesis of melatonin. In the rat pineal gland, several miRNAs were shown to be preferentially expressed relative to other organs or parts of the CNS [39]. While one of the enriched miRNAs, *miR-125b*, did not show substantial day/night differences, cycles of moderate amplitude were observed in a number of other cases, such as *miR-143* and *miR-124*. Because of corresponding findings in the retina, particular attention was paid to a polycistronic miRNA cluster, *miR-183-96-182*. However, the three miRNAs were expressed to a highly different extent. *miR-182* amounted to over 28% of total miRNA extracted. Nevertheless, all of them exhibited circadian rhythms with a maximum/minimum ratio of about two. The maxima of *miR-182* and *miR-183* occurred during photophase, whereas *miR-96* showed a sharp rise after light onset, from where its concentration steadily dropped towards a minimum at the end of scotophase. With regard to expectable reductions in stability and the expression patterns of target mRNAs, these patterns would be line with a mainly nocturnal metabolic activity of the pineal gland. However, the much lower circadian amplitudes of these miRNAs compared to that of melatonin biosynthesis indicate that their contribution to the melatonin rhythm is either minor or non-existent.

Another miRNA, *miR-483*, was shown to directly target the mRNA for aralkylamine *N*-acetyltransferase (AANAT), the primarily rate-limiting enzyme of melatonin formation [39]. As with many miRNAs, reporter constructs indicate that the binding site is present in the 3'-UTR of the mRNA. Again, the circadian amplitude of *miR-483* was rather moderate, but, contrary to most other miRNAs studied, a strong decline in its abundance was observed during ontogeny. Transfection of neonatal pinealocytes with a *miR-483* antagonist caused a substantial increase in melatonin synthesis [39]. Thus, *miR-483* may be partially or fully responsible for low melatonin formation in the pineals of neonates, and its decreasing expression seems to allow the developmental rise of the hormone.

Transcripts of the *miR-183-96-182* cluster were also detected in the zebrafish pineal gland [40]. In this species, as in many other fish, the pineal acts also as a circadian pacemaker. In accordance with this additional role, various core or accessory oscillator and light-input genes were light-induced, but this was also observed for the three miRNAs [40]. Importantly, *miR-183* was shown to target not only the mRNAs of E4BP4-6 (=NFIL3-6), a clock-controlled transcription factor that feeds back to the oscillator, but also that of AANAT2, via binding sites in the respective 3'-UTR regions. Using reporter constructs, the interaction of *miR-183* with the *Aanat2* 3'-UTR was shown to reduce the expression by about one half, an effect abolished by mutations in the 3'-UTR [40]. Thus, *miR-183* does not only interact with the *Danio rerio* oscillator, but also decreases melatonin formation under conditions of light exposure.

Another aspect concerning mRNA stability and usage became evident in the *Xenopus* retina, which bears, in the photoreceptor cells, an autonomous circadian oscillator system that controls melatonin synthesis and secretion in this part of the eye. While searching for clock-controlled genes and their products, a deadenylase belonging to the CCR4 family was discovered that was strongly up-regulated during scotophase and, therefore, named nocturnin (NOC) [41,42]. With regard to the temporal coincidence of melatonin formation and NOC expression during the night, one should not expect a nocturnal reduction of, e.g., *Aanat* mRNA availability by poly(A) decay, but instead, NOC seems to be required for oscillator output functions and, thereby, contributes to circadian physiology. As will be discussed in Section 4 and Section 6, mammalian NOC homologs are also expressed in various other organs and cell types, where they display substantial effects in metabolism control and differentiation, thereby overlapping with effects of melatonin.

Circadian periodicity of gene expression is not restricted to mRNAs and their modulation by miRNAs and deadenylases. With regard to the quantitative importance of lncRNAs [23], which has become evident during recent years (*cf.* Introduction), high-amplitude rhythms in their abundance should no longer be a surprise. However, the remarkably high number of cycling long noncoding transcripts has been rather unexpected. The quantitative importance of circadian lncRNA expression has been impressively demonstrated in the rat pineal [43]. Comparisons of day/night differences revealed differential expression of 112 lncRNAs. About half of them were preferentially expressed during the night. Over 100 displayed maximum/minimum ratios above two. In some cases, the amplitudes were remarkably high (night/day ratios: *lncSN001*-267; *lncSN004*-178; *lncSN012*-50; *lncSN081*-36; *lncSN215*-0.2). Several selected lncRNA rhythms were shown to persist in constant darkness (DD). The rhythms of *lncSN001* and *lncSN016* clearly depend on the suprachiasmatic nucleus (SCN) because they were abolished by surgical decentralization of superior cervical ganglia and their expression was stimulated by isoproterenol. Moreover, several selected lncRNAs were upregulated by dibutyryl-cAMP in cultured pineals. Light at night (LAN) suppressed nocturnal levels of several lncRNAs tested [43]. Although the precise function of these numerous cycling lncRNAs is not yet known, these findings are of presumably fundamental importance and shed light on the necessity of further studying and analyzing the roles of these noncoding transcripts. Without their consideration, our future understanding of pineal physiology and the transmission of intracellular circadian signals would remain incomplete and inaccurate.

## 3. Epigenetic Modulation of Melatonin Receptor Expression—Initial Findings

The modulation of melatonin’s actions by epigenetic mechanisms is, with high likelihood, not restricted to rhythmic biosynthesis in melatonin-producing organs, but should also modulate receptor expression in the hormone’s target cells. To date, this aspect has not yet been systematically studied, but a few initial findings indicate the existence of epigenetic control at this level of action. In C6 glioma cells, experiments using valproic acid showed that changes in the expression of the melatonin receptor gene *MTNR1A* (coding for receptor MT_1_) were accompanied by alterations in the mRNA levels of methyl CpG binding protein 2 (MeCP2) and of histone deacetylases, HDAC1, 2 and 3 [44], indicating that both the DNA methylation pattern and chromatin remodeling via histone deacetylation may be associated with changes in MT_1_ expression. This conclusion was supported by an up-regulation of MT_1_ observed after treatment with the HDAC inhibitor trichostatin A, which is structurally unrelated to valproic acid [44].

Another line of evidence concerns the influence of a piRNA on *MTNR1A* expression [45]. piRNAs, named because of their binding to PIWI proteins, have previously been thought to be mainly involved in transposon silencing, maintenance of germ-line integrity and gonad functions. Although the high number of known piRNAs, which exceed 30,000 in humans, may already be indicative of additional functions, their precise roles outside the germ line are largely unknown. In the study mentioned, a non-gonadal function of *piR_015520* became evident by its expression in the brain, in addition to that in testes [45]. In transfected HEK 293 cells, *piR_015520* down-regulated the expression of *MTNR1A*. The sequence of this piRNA is located in intron 1 of the *MTNR1A* gene. However, it is not known whether the observed effect is caused by piRNA-directed protein binding to the receptor gene or piRNA-mediated RNA silencing comparable to actions of RNA-Induced Silencing Complexes (RISC). Since *piR_015520* did not interact with PIWI protein in an electrophoretic mobility shift assay (EMSA), these classic interaction partners of piRNAs can be excluded. Instead another, not yet identified protein was shown to bind to *piR_015520* [45].

## 4. Circadian Oscillators and Epigenetics—A Role for Melatonin?

In the context of circadian oscillations, a relatively large body of information exists on miRNAs, lncRNAs and proteins involved in mRNA stability and chromatin remodeling (Table 1). In most cases, a direct relationship to melatonin has not been investigated. However, the hormone’s influence on both central and peripheral circadian oscillators [37] indicates that its actions should be interrelated to a certain degree with these aspects of epigenetics and the circadian fine tuning of gene expression.

**Table 1:** Circadian aspects of miRNAs, lncRNAs and proteins involved in chromatin remodeling. Findings discussed in Section 2, Section 6 and Section 8 are omitted. ^1^ ICR = imprinting control region; ^2^ Mico = maternal intergenic circadian oscillating; NAMPT = nicotinamide phosphoribosyltransferase; os = opposite strand; ^3^ ZT = Zeitgeber time; ^4^ PSF = polypyrimidine tract-binding protein-associated splicing factor; ^5^ NONO = non-POU domain-containing octamer binding protein; ^6^ SFPQ = splicing factor proline/glutamine-rich.

Organism/Tissue or Cells	Main Findings	References
Mouse/SCN	Circadian rhythms of *miR-219* and *miR-132*; *miR-219*; knockdown lengthens circadian period; *Cry1*/*Cry2* double knockout abolishes rhythms of *pre-miR-219-1* and *miR-219-1*; CLOCK/BMAL1 over-expression stimulates *pre-miR-219-1*; *miR-132* is induced by light (also by LAN) via ERK/MAPK, but acts as negative regulator of photic entrainment; *miR-132* is presumably target of CBP (CREB binding protein)	[46,47]
Rat/primary cortical neurons	*miR-219* over-expression suppresses NMDA-induced Ca^2+^ influx	[46]
Mouse/brain and P19 cells	*miR-219* down-regulates NMDA signaling by targeting *CamkII* subunit γ mRNA	[48]
Mouse/SCN	*miR-132* targets mRNAs of proteins involved in chromatin remodeling (*Mecp2*, *Ep300*, *Jarid1a*) and translational control (*Btg2*, *Paip2a*); MeCP2 binds to *Per1* and *Per2* promoters; BTG2 and PAIP2A enhance decay of *Per1* and *Per2* mRNAs	[49]
Mouse/retina	Circadian rhythms of 16 lincRNAs	[50]
Mouse/retina	Circadian rhythms of 12 miRNAs, including those from *miR-183-96-182* cluster	[47,51]
Mouse/brain	Two overlapping imprinted ncRNAs from intergenic region Dlk1–Gtl2 that contains an ICR ^1^ are exclusively expressed at maternal chromosome, from both strands: *Mico1* and *Mico1os* ^2^; both oscillate in a circadian fashion	[52]
Mouse/hypothalamus, hindbrain, forebrain, cortex, hippocampus, cerebellum; neurons but not glia	Transcript of Prader–Willis locus *SNORD116* is spliced into multiple snoRNAs and the lncRNA *116HG*; *116HG* forms subnuclear clouds that increase postnatally and are associated with large-scale chromatin decondensation; size of clouds smaller at ZT16 ^3^ than at ZT6; in *SNORD116*^−/−^ mice, expression of 6467 genes is altered at ZT6, of 3240 genes at ZT16; relative to WT, *Clock*, *Cry1* and *Per2* are up-regulated at ZT6, *Cry1*, *Cry2* and *Per1* down-regulated at ZT16	[53]
Mouse/serum	Circadian rhythms of *miR-152* and *miR-494*; circulating miRNAs may influence oscillators via microvesicles	[54]
Human/HEK293 cells	*Bmal1* is targeted at 3'-UTR by *miR-494* and *miR-142-3p*	[54]
HTC116, HT29 and NIH3T3 cells	*miR-192/194* cluster targets *Per1*, *Per2*, and *Per3* and alters circadian rhythms	[55]
Mouse/liver	Circadian rhythms of 85 miRNAs; several miRNA/mRNA target pairs identified, including core oscillator mRNAs; *miR-181d* and *miR-191* are inversely correlated with *Clock*/*Bmal1* and presumably involved in peripheral clocks; *miR-328* and *miR-383* positively correlated with *Per1*/*Cry1*	[47,56]	
Mouse/liver	REV-ERBα drives *miR-122* transcription; knockdown of *miR-122* alters expression of hundreds of hepatic mRNAs	[57]	
Mouse/liver	*miR-122* targets *Noc* mRNA; *miR-122* knockdown increases the amplitude of the nocturnin rhythm	[58]	
Mouse/various organs	*Noc* mRNA is rhythmic in several brain regions, retina, heart, kidney, spleen, and liver	[59,60]	
Mouse/liver	Several hundred mRNAs exhibit circadian rhythms in poly(A) tail length, even in cases in which mRNA levels are not rhythmic	[61]	
Mouse/liver	CLOCK controls rhythmic transcription of *Noc*; *Clock* mutants exhibit dampened *Noc* rhythms	[62]	
Human/Huh7 hepatoma cells	Binding of CLOCK/BMAL1 to E-box in *Noc* promoter	[63]	
Mouse/liver	NOC stabilizes *iNOS* mRNA; NOC deficiency blunts the nocturnal peak of *iNOS* mRNA	[33]	
Mouse/liver	Circadian rhythms of 54 miRNAs, 16 lincRNAs and several antisense transcripts, including a *Per2* antisense RNA (*asPer2*); rhythms in histone modifications: especially, H3K4me3, but also H3K4me1, H3K9ac, H3K27ac (at active enhancers), and H3K36me3 enriched in actively transcribed genes	[64]	
Mouse/embryo fibroblasts, liver	SIRT1, an accessory circadian oscillator protein, histone deacetylase and aging suppressor, promotes PER2 degradation by deacetylation, is required for high amplitudes of *Per2*, *Cry1*, *Bmal1* and *ROR*γ transcription rhythms, is recruited to the BMAL1/CLOCK complex and controls the expression of E-box-containing genes such as *Per2*, *Cry1* and *NAMPT* via cycling NAD^+^ concentration	[65,66,67,68,69]	
Mouse/liver, lung, fibroblasts	PER proteins form complexes that include PSF ^4^, which recruits the scaffold SIN3A associated with a HDAC that rhythmically suppresses *Per1* transcription by deacetylating histones at the promoter	[70]	
Mouse/liver, 3T3 cells	NONO ^5^ interacts with PER1 and modulates its activity	[71]	
Mouse/liver, brain areas incl. SCN	NONO associates with PER1 or PER2 at *Rev-erbα* and *Dbp* promoters; NONO couples the oscillator to cell cycle; NONO also interacts with ncRNAs	[72,73]	
Rat/GH4C1 cells	NONO and SFPQ ^6^ induce chromatin remodeling at prolactin promoter and couple *Prl* expression to circadian oscillator; NONO/SFPQ over-expression decreases promoter activity and disrupts circadian *Prl* rhythm	[74]	

Direct effects of melatonin on oscillator gene expression and other factors mentioned in Table 1 have been reported in a few cases. As summarized elsewhere [37], melatonin was shown to phase-shift the *Rev-erb*α rhythm in the SCN and other organs, required for a robust amplitude of this accessory oscillator component, with secondary effects on the expression of *Bmal1*, *Cry1* and *Per1*. In the peripheral clock of the mouse adrenal cortex, melatonin is required for high amplitudes of PER1, CRY2 and BMAL1 rhythms [75]. Another action concerns the accessory oscillator protein SIRT1, an important player in the maintenance of rhythm amplitudes in peripheral clocks. In several studies, melatonin was reported to up-regulate SIRT1, such as in the senescence-accelerated mouse strain SAMP8 [76], in the hippocampus of sleep-deprived rats [77], and in neuronal cultures from aged rats [78]. In the latter case, enhanced deacetylation of the SIRT1 substrates PGC-1α, FoxO1, NF-κB, and p53 was also observed. At first glance, these findings seem to be at variance with other results on SIRT1 suppression obtained in tumor cells, as will be discussed in Section 8. Interpretations of these seemingly contradictory data have to consider the differences between normally oscillating cells and tumor cells with impaired clocks due to silenced oscillator genes with tumor suppressor function. Nevertheless, these results also show profound influences of melatonin on the deacetylase SIRT1 and its downstream effects including those on local chromatin structure.

As summarized in Table 1, circadian oscillators are multiply involved in epigenetic processes. A specific aspect concerns the involvement of the core oscillator component CLOCK, which acts not only as an E-box-binding protein, but also as a histone acetyltransferase (HAT). SIRT1, which associates with the CLOCK/BMAL1 complex depending on a cycle of its coenzyme NAD^+^, is a histone deacetylase. These two enzymes, which act antagonistically at histones, are notably also involved in important processes within the oscillator and in the control of output functions [68,69]. CLOCK has been shown to acetylate nonhistone proteins, in particular, its interaction partner BMAL1. SIRT1 deacetylates various nonhistone proteins, including BMAL1, PGC-1α, FoxO1, NF-κB, and p53. The active CLOCK/BMAL1/SIRT1 complex induces the transcription of numerous genes with E-box-containing promoters. Among these, NAMPT is of crucial importance because its activity drives the NAD^+^ cycle [65,66,67,68,69]. Via NAD^+^ availability to SIRT1, this cycle allows transcriptional rhythmicity even though SIRT1 and CLOCK are virtually arrythmically expressed. The presence of E-boxes in a large number of circadian controlled genes (CCGs) implies that the epigenetic regulation by and within the oscillator influences the expression of numerous proteins. Therefore, any modulation of central or peripheral oscillators by melatonin should result in a plethora of effects.

Prolactin expression represents a specific example in which melatonin is known to modulate a circadian output function. In this case, the expression is also influenced by other chromatin remodeling factors such as NONO and SFPQ. Most information is available from nocturnally active rodents, in which prolactin secretion in inhibited via the MT_1_ receptor [79,80]. However, this seems to be entirely different in diurnally active species. In humans, the circadian rhythms of melatonin and prolactin are approximately in phase [81].

A puzzling question that deserves further clarification is that of the similar phasing of melatonin and nocturnin. At first glance, one might be inclined to assume a synergism, but this is not necessarily the case. In particular, their actions on *iNOS* expression are obviously antagonistic, at least under conditions studied, since melatonin was shown to down-regulate iNOS [82,83,84] (further details in [1]). It may be that this effect by melatonin is mainly observed under high-grade inflammatory conditions, whereas that by NOC is primarily important under basal rhythmic conditions. The support of *iNOS* mRNA stability by NOC requires further elucidation, as to the alternative of either an additional action independent of deadenylase activity or preferential deadenylation of an ncRNA that targets *iNOS* mRNA. The first possibility is not beyond reality because a *Noc* mutant deficient of deadenylase activity was still capable of stimulating the translocation of PPARγ (peroxisome proliferator-activated receptor-γ) to the nucleus [85]. Opposite effects of melatonin and NOC at night might mutually limit the respective actions. This may be also assumed for other reasons, e.g., the lowered body temperature of *Noc*^−/−^ mice [85], and various contrary actions concerning energy expenditure and lipid metabolism (see Section 6).

## 5. Light at Night—Epigenetic Changes during Chronodisruption and Melatonin Shutdown

The circadian oscillator system and melatonin levels are both affected by perturbing light signals at night. The clocks may be reset according to the phase-response curve, while melatonin synthesis and secretion are precipitously turned off by LAN [86,87,88]. These changes are of particular importance with regard to shiftwork, which has been associated with numerous diseases and disorders [35,89,90,91,92,93,94]. This health risk has been discussed many times and a full record would exceed the scope of this article. Although the dual effects of LAN have been recognized and addressed, it remains difficult to causally distinguish between the intertwined contributions of a perturbed oscillator and the melatonin shutdown. This is also the case in animal studies on light-induced changes in the SCN, which are mainly focused on circadian entrainment, but may also be of importance to secondary changes in the pineal by LAN. In the mouse SCN, brief light pulses cause phosphorylation of the transcriptional modulator MeCP2, trimethylation of histone H3 and acetylation of histone H2A.Z, events that lead to transactivation of light responsive genes such as *Per1*, *Per2*, *Btg2* (*cf.* Table 1) and also *pre-miR-132* [49]. The upregulation is terminated via two mechanisms, (i) by RISC formation involving the processed *miR-132* [46,49]; and (ii) by enhanced mRNA deadenylation as a consequence of BTG2 binding to the CAF1/CCR4 deadenylase complex [49]. These events in the SCN are transmitted to the pineal gland in terms of resetting, but should also contribute to the photic shutoff of melatonin formation. In the rat pineal, the mainly SCN-dependent circadian rhythms of numerous lncRNAs were affected by LAN, which caused rapid decreases (*t*_1/2_ values between 9 and 32 min) in several lncRNA species [43].

In humans, there is still an unfortunate paucity of direct evidence for gene-specific epigenetic changes as a consequence of LAN or of the light-induced reduction of melatonin. Most pertinent publications summarize hints derived from studies on epigenetic changes within normal circadian cycles or on dysregulations observed in the manifest diseases. Nevertheless, the few reports on effects by LAN may encourage following this experimental route further. Direct evidence for epigenetic alterations because of long-term shiftwork was obtained in a study based on archived human DNA samples from whole blood [95]. In this material, the *Cry2* promoter was found to be hypermethylated, whereas the *Clock* promoter was hypomethylated, two changes also present in breast cancer. Hypermethylations were also detected in a cancer-relevant histone deacetylase gene, *Hdac2*, and in the *Mdb2* (methyl-CpG-binding domain 2) gene, which encodes a major methylation-related factor with transcriptional repressor function. A genome-wide DNA methylation analysis revealed widespread alterations: 3593 CpG sites were hypermethylated and 1816 CpG sites hypomethylated in long-term shiftworkers [95]. Corresponding findings on site-specific increases or decreases in DNA methylations in long-term shiftworkers were obtained in another, smaller study, which, however, focused on imprinted genes. Significant hyper- or hypomethylations were detected in 20 and 30 CpG sites, respectively, in the promoter regions of genes that are normally subject to imprinting [96]. In an experimental setting using 4T1 breast cancer cells inoculated into BALB/c mice, tumor growth was favored by LAN, along with changes in the DNA methylation pattern, effects that were partially reversed by melatonin [97].

## 6. Metabolic Disorders and Energy Expenditure, Melatonergic Counteractions and Epigenetic Regulation

The significance of epigenetic modulation of metabolism as well as the consequences of epigenetic dysregulation for the acquisition of metabolic diseases has gained increasing attention [12,13]. With regard to melatonin, various data exist concerning actions against obesity, metabolic syndrome, diabetes type 2, cardiovascular diseases, and bone adipocyte differentiation [1,35,36,37,98,99,100,101,102]. Again, these diseases and disorders are related to circadian malfunction and pertubations by LAN. However, the role of epigenetics in melatonin-induced normalizations is still an emerging field, which does not yet cover all aspects of ameliorations with melatonin. Nevertheless, initial findings are encouraging. Moreover, compelling evidence exists for strong epigenetic effects in this area by other factors that favor the development of metabolic disorders. Known counteractions by melatonin likely interfere with these changes or may reverse them.

One of the multiply involved players in metabolic regulation is PPARγ. As summarized elsewhere, also in its relation to melatonin [101], it is an important factor in the control of energy balance, mitochondrial proliferation and electron flow, insulin sensitivity, differentiation of mesenchymal stem cells, as well as various other processes including suppression of overshooting inflammation and mechanisms of neuroprotection. The multiplicity of actions implies, necessarily, several complications concerning cell specificity, differences between subforms, and modulation by upstream factors. These include AMP kinase (AMPK), nitric oxide, and SIRT1, which act via the PPARγ coactivator-1α (=PGC-1α) [103]. Moreover, *Pparγ* was shown to exhibit a circadian rhythm not controlled by the SIRT1/AMPK route, but rather by DBP and E4BP4 (=NFIL3), which bind to an exonic D-site [103]. Any influence of melatonin on a circadian oscillator driving *Ppar*γ expression should have the potential of modulating this important regulator, but, under basal oscillatory conditions, such an effect can be expected to be phase-dependent. As mentioned above, the mRNA of an E4BP4 subform is targeted by *miR-183*, in parallel to that of AANAT2. Moreover, the *Dbp* promoter is influenced by NONO (*cf.* Table 1).

Contrary to its upstream factor PGC-1α, PPARγ was shown to respond to melatonin [104]. In differentiating human mesenchymal stem cells (hMSCs), melatonin down-regulated PPARγ, an effect associated with the suppression of adipogenic in favor of osteogenic differentiation. However, two studies on the conversion of 3T3-L1 preadipocytes to adipocytes led to results conflicting with each other, both with regard to PPARγ expression and adipogenic differentiation [105,106]. More recently, hepatic adiposity was reported to be decreased by melatonin [107]. However, the mechanism may be different from that observed under conditions of differentiation, and other antiadipogenic actions of melatonin also require further detailed elucidation [94,108,109,110]. Nevertheless, melatonin appears to be a predominantly antiadipogenic agent, which also corrects deviations by LAN that include epigenetic alterations mentioned above. Moreover, melatonin-induced browning of fat cells [109] implies mitochondrial proliferation, but it is uncertain as to whether the otherwise adipogenic PGC-1α/PPARγ pathway may be involved. The consideration of melatonin as a regulator of fat metabolism strongly indicates, again, an antagonism to another epigenetic factor, NOC. This protein has turned out to be adipogenic in multiple ways, by stimulating intestinal lipid uptake [31,111,112,113], adipogenesis [31,85,114] and lipid storage [113]. In addition, NOC seems to be involved in the regulation of glucose homeostasis and insulin sensitivity and may promote insulin resistance [113]. NOC was also shown to enhance PPARγ activity [114] and to promote the nuclear translocation of this factor [85]. On the other hand, PPARγ is a negative regulator of osteogenesis [114,115,116]. Accordingly, osteogenesis was shown to be also antagonized by NOC, at the expense of adipogenesis [31,114,115,116], with important consequences for bone loss in metabolic disorders and during aging. Both PPARγ and NOC down-regulated a major osteogenic factor, IGF-1 [115,117]. The deadenylase activity of NOC was identified as being decisive by targeting an *igf1* mRNA subform containing a long 3'-UTR [117]. Again, the shift in the balance between adipogenesis and osteogenesis reveals an antagonism between NOC and melatonin, because the latter acts clearly osteogenic, effects involving activation of MAPK and Wnt5 signaling, and induction of Runx2, osteocalcin as well as bone morphogenic protein (BMP)-2 and -4 [104,118,119,120,121]. Epigenetic effects of melatonin in favor of osteoblast differentiation have not yet been directly studied, but other investigations have shown that PPARγ signaling is affected. From a certain stage on, DNA hypermethylation in the C/EBPα (CCAAT/enhancer binding protein α) promoter prevents PPARγ binding, while histone acetylation is reduced by HDAC1 [122]. Moreover, lysine demethylase 6A (KDM6A) has been found to be decisive for osteogenesis, whereas formation of H3K27me3 by the histone methyltransferase EZH2 (enhancer of Zeste homology 2) favors adipogenesis, as demonstrated by respective inhibitor and knockdown experiments [123]. To what extent NOC and melatonin are also acting via these mechanisms, remains to be studied. Nevertheless, knockout of the *Noc* gene has revealed an impressive spectrum of metabolic and developmental changes. In *Noc*^−/−^ mice, bone formation is enhanced, lipid uptake and storage is reduced, glucose homeostasis and insulin sensitivity are altered, and animals are resistant to liver steatosis and diet-induced obesity [30,31,114,116,124,125]. Since *Noc*, contrary to other deadenylases, possesses properties of an immediate early gene [126], one might, at first glance, think primarily of short-term responses, which rapidly modulate the stability of target mRNAs as well as PPARγ translocation and binding. However, its importance has been also demonstrated in long-term studies using mice fed a high-fat diet over four generations [127]. These animals did not only show a persistent obese phenotype, but also had steadily elevated NOC levels. It may be an intriguing idea to test whether melatonin is capable of reprogramming such animals by epigenetic mechanisms.

## 7. Epigenetics of Inflammation and Oxidative Stress *vs.* Antioxidant Properties of Melatonin

**Figure 1:**
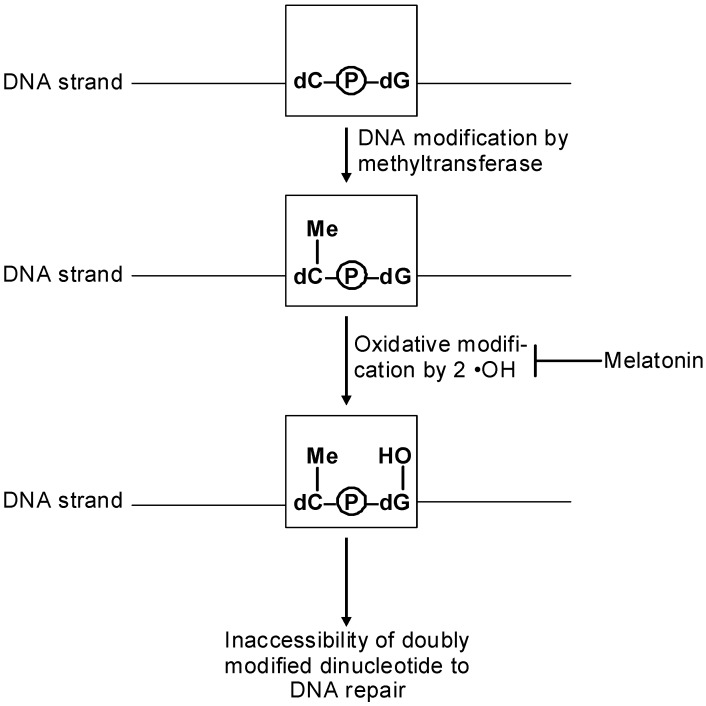
Prevention of 8-hydroxy-deoxyguanosine formation by antioxidant actions of melatonin may help avoid DNA repair failure in CpG islands. The primary hydroxylated guanine spontaneously turns into the oxo tautomer (not shown in the figure).

Oxidative or nitrooxidative stress can have different causes, such as mitochondrial dysfunction, activation of NAD(P)H oxidases, inflammation, or exposure to prooxidant toxins. Melatonin is a potent antioxidant and antinitrosant agent [2,5,82,83,84,128,129]. However, these effects are sometimes conditional or tissue-specific. While melatonin is known to antagonize the activation of neuronal NO synthase (nNOS) in the central nervous system, it was shown to up-regulate nNOS expression in HaCaT keratinocytes [130,131]. The quantities of NO formed were sufficient to decrease mitochondrial membrane potential and oxidative phosphorylation and may serve as a signal connecting the circadian system to mitochondrial function [131]. With regard to the immune system, melatonin’s actions are conditional, either in an antiinflammatory, antioxidant and antinitrosant fashion, but alternately in an immune-stimulatory, proinflammatory and, thus, prooxidant way [6,101]. Mitochondrial malfunction and oxidative stress resulting from low-grade inflammation are observed, e.g., during aging, changes that are partially counteracted by melatonin [101]. In recent years, numerous epigenetic changes related to aging have become known [132,133,134,135]. However, relationships to melatonin are only exceptionally discernible, although this might be a promising area for future research. In general, overall DNA methylation decreases during senescence, whereas hypermethylation is observed in specific loci, e.g., in the *c-fos*, *igfII*, and *p16ink4a* genes [132]. Changes in histone modification are frequent and lead to chromatin alterations. For example, two histone methylation complexes, PRC1 (polycomb repressive complex member Bmil1) and PRC2 (polycomb repressive complex member EZH2), are typically reduced, whereas the histone demethylase JMJD3 (jumonji domain containing 3) is up-regulated [132]. Consequently, reductions of methylated histones are usually detected, particularly of H3K36me3, H3K9me3 and H4K20me. The acetylated histone H4K16ac is increased, and, correspondingly, the histone deacetylase SIRT1 is reduced [132]. This decrease of SIRT1 may be corrected by melatonin, as indicated by the few findings obtained in the context of aging [76,78], results that contrast, however, with changes observed in melatonin-treated cancer cells. Notably, senescence-associated heterochromatin foci (SAHFs) can be used as markers of advanced aging [132]. Vascular and cardiac aging has been related to increases in *miR-29* and *miR-34a* [132,133,134]. The latter was shown to target the 3'-UTR of *Pnuts* mRNA and, thereby, to age-dependently reduce the level of PNUTS protein [134], *i.e.*, the protein phosphatase 1 nuclear targeting subunit required for the transfer of the phosphatase to the nucleus. Importantly, this effect was also observed upon myocardial infarction [134], with consequences to ROS (reactive oxygen species) formation, DNA damage and apoptosis, findings of great interest with regard to melatonin’s known cardioprotective and antiapoptotic effects [135,136,137]. In endothelial cells, ROS were also found to induce members of the *miR-200* family, in particular, *miR-200c* [138], which down-regulate the transcription factor Zeb1 (zinc finger E-box binding homeobox), an effect also associated with apoptosis and senescence [132,138]. An additional epigenetic aspect of ROS-induced changes concerns the formation of 8-hydroxy-dG (8-oxo-dG) in CpG islands. If the cytosine is already methylated, this second modification prevents DNA repair and may promote amyloid deposit formation in the brain [132]. Again, melatonin can be beneficial in this context, since it was shown to reduce 8-hydroxy-dG [139,140] (Figure 1). It may also be noted that several lncRNAs are involved in the DNA damage response and respective actions of p53 [23]. Further modifications by ROS are related to inhibition of histone deacetylation. In the cardiovascular system and in the lungs, the oxidants cause a decrease in SIRT1 or in HDAC2, respectively [132]. In the latter case, this results in NF-κB up-regulation and initiation of an inflammatory response.

The roles of NF-κB subforms have been also addressed with regard to antiinflammatory and antioxidant actions of melatonin [5,36,141,142]. Initially, melatonin was found to inhibit NF-κB actions [143] and binding to the DNA [144,145]. The decrease of binding was related to the acetylation of p52, the subunit of an NF-κB subform [144]. NF-κB is also regulated by the coactivator complex CBP/p300 (CBP = CREB binding protein), which possesses histone acetylase (HAT) activity and an RNA polymerase II binding site. Inhibition of NF-κB binding has been reported to be causal to the down-regulation of iNOS and COX-2 (cyclooxygenase 2) by melatonin [5,144]. Similar assumptions have been made for other melatonin-mediated antiinflammatory effects, such as suppression of proinflammatory cytokines and matrix metalloproteinases [5]. From a conventional point of view, one might interpret these effects via the classic melatonergic signal transduction pathway, the decrease of cAMP, which would reduce CREB phosphorylation and diminish the CBP/p300 coactivator of NF-κB. To which extent additional effects, such as direct inhibition of p300 HAT activity and NF-κB acetylation or melatonin-mediated recruitment of HDACs, contribute to the balance remains to be clarified.

Apart from the suppression of inflammation and various other mechanisms [128], antioxidative protection by melatonin involves the up-regulation of antioxidant enzymes. A particular complication results from the fact that induction of these enzymes requires an increase rather than a decrease of NF-κB. As discussed elsewhere [5], the subforms of this transcription factor may act differently. The stimulation of antioxidant enzymes may not be mediated by the classic p50:p65 dimer, but instead by RelB:p52. However, this problem is not fully solved, because p52 acetylation was reported to be inhibited by melatonin [144].

The up-regulation of antioxidant enzymes may be explained by actions of another player, Nrf2 (nuclear factor erythroid 2-related factor 2), which is up-regulated by melatonin [5]. Nrf2 was also shown to be acetylated by CBP/p300, but, again, another melatonin effect, the decrease in cAMP, is not immediately compatible with an enhanced CBP/p300 HAT activity. The control of antioxidant enzymes and redox balance by epigenetic modulation is worth further investigation and presumably much more complex than believed to date, which will become obvious as soon as ncRNAs come into play.

## 8. Melatonin, Cancer and Epigenetics

Cancer research is that field in which the importance of genome-wide epigenetic alterations has become most impressively evident, also in quantitative terms of findings (for recent reviews see refs. [9,146,147,148,149]). Changes are observed at all levels of epigenetic mechanisms, including DNA methylation patterns, aberrant expression of miRNAs [146] and other ncRNAs [150], as well as changes in histone modification [148]. The deviations concern large-scale DNA hypomethylation, the appearance of large blocks of aberrant histone modifications [146], derepression of numerous genes that favor the neoplastic phenotype such as cancer-promoting genes and oncogenes [148], and inactivation of tumor suppressor genes by promoter hypermethylation [146,148]. However, the changes observed are of a highly dynamic nature. They start with epigenetic alterations associated with chronic inflammation or persistent viral infection [149], and they continue during cancer progression, thereby contributing to cancer plasticity [147]. The *trans* acting lncRNA *HOTAIR* was found to drive metastasis and to be a prognostic marker of poor survival [27,151]. The deviations in DNA methylation patterns may even be subject to further enzymatic modifications by TET (ten-eleven translocation) family proteins which convert 5-methylcytosine (5-mC) to 5-hydroxymethylcytosine (5-hmC) [152]. To date, it is known that TET1 expression and 5-hmC abundance have been reduced in several tumors and hematopoietic malignancies tested [153]. Although these findings strongly suggest a cancer-antagonizing role of 5-hmC, the consequences remain to be further elucidated. Moreover, many earlier data on 5-mC profiles have to be re-examined, because conventional techniques did not distinguish between 5-mC and 5-hmC.

The plasticity of the epigenetic system raises the question of the respective transitions between pre-cancerous and malignant states. The idea had been forwarded that epigenetic alterations, which are, in fact, induced by a stress response (*cf.* [154,155,156]), may turn under conditions of accumulated stress an adaptive, basically advantageous process into pathological deregulation [157]. If this assumption is valid, stress-reducing properties of melatonin [158,159,160,161] should be of interest and may be reflected by attenuated stress patterns of the epigenome. With regard to cancer, these possible relationships should not be solely seen from the viewpoint of cell stress, but rather in a systemic way, because the effects of stress are manifold and involve secondary responses of the nervous, endocrine and immune systems.

Another field in which pre-cancerous epigenetic changes can favor malignancy is that of shiftwork and LAN. As outlined in Section 5, it is difficult to distinguish between perturbations of circadian oscillators and the photic shutoff of melatonin synthesis. However, the disturbance of clock functions is also related to melatonin, which both can reset oscillators and is controlled by them. Long-term shiftwork with frequently repeated exposure to LAN was shown to cause extensive changes in DNA methylation patterns, including cancer-relevant [95,96], imprinted [96], and oscillator genes [95] as well as in histone modification [95]. Promoter hypomethylation in the oscillator gene *Clock* and promoter hypermethylation of *Cry2* [95] represent changes also detected in breast cancer. As summarized elsewhere [37], several core oscillator components act as tumor suppressors, and various tumors and tumor cell lines exhibit hyper- or altered methylation in the promoters of *Per1*, *Per2*, *Per3*, *Cry1*, *Cry2* and *Bmal1*. These changes in the promoters may be regarded as a necessity for tumor cells to maintain their transformed state and to escape from circadian gating of the cell cycle. Concerning cancer, the role of the *Clock* gene is, however, obviously different. The CLOCK protein, which also exhibits HAT activity, seems to have tumor-promoting properties and to favor cell proliferation. Therefore, its enhanced expression because of hypomethylation should be of advantage for a tumor cell. However, all interpretations of these findings have to consider that the changes in the core oscillator genes make the circadian clocks of tumor cells dysfunctional. Therefore, any reversal by an epigenetically acting regulator molecule hits these genes in a strongly deviating, perhaps poorly- or even non-oscillatory state, which makes a fundamental difference to the cyclicity in the nontransformed cell. As indicated by initial findings, melatonin may be such a molecule, which causes profoundly different effects in nontumor and tumor cells, *i.e.*, under conditions of a normally operating or a dysfunctional circadian oscillator.

In animal models, melatonin did not only reverse adverse effects by LAN including tumorigenesis [93], but also reduced the growth rates of breast tumors and changed the global DNA methylation pattern to a presumably favorable state [97]. However, various modes of action have been discussed to contribute to the oncostatic and other anti-tumor effects of melatonin.

A specific aspect of epigenetic changes in tumors concerns the complicated relationship between melatonin and sirtuins. On the one hand, sirtuins have not only been seen in their first-discovered role as aging suppressors, but also as guardians of the genome [162]. On the other hand, with regard to cancer, their actions are not at all uniform. While the SIRT2 and SIRT6 actually appear to be tumor suppressors, the most frequently studied subform SIRT1 seems to conditionally operate either as a tumor suppressor or as cancer promoting factor [163]. Since SIRT1 is an accessory oscillator protein [164] required for high amplitudes in the circadian transcription of *Per2*, *Cry1*, *Bmal1* and *ROR*γ (further details see ref. [37]), the conditionality has, again, to be interpreted on the basis of strongly oscillatory *vs.* poorly-/non-oscillatory states. As a participant of a robustly oscillating machinery, SIRT1 should contribute to the overall antitumorigenic activities of circadian clocks, which are responsible for a daily, cell- and time-specific chromatin remodeling. This should be different in a more or less arrested state of the oscillator, in which some components are steadily over-, others under-expressed. Deacetylation of histones and other proteins, if not imbedded into cyclicity, may, in fact, be detrimental. Notably, a transcriptional master regulator, DBC1 (Deleted in Breast Cancer 1), negatively controls the deacetylases SIRT1 and HDAC3 [165]. Moreover, the findings that both the histone acetylase CLOCK and the histone deacetylase SIRT1 can display tumor-promoting properties appear, at first glance, implausible, but this contradiction can be solved by considering the differences between a normally oscillating clockwork and deregulated expression of its components. In fact, SIRT1 was found to be over-expressed in prostate cancer tissue [166]. The observation that the loss of melatonin can also lead to circadian deregulation gave rise to the idea it might correct SIRT1 activity [167,168]. Thereafter, melatonin was shown to exert an antiproliferative effect in human prostate cancer cell lines and in a mouse prostate adenocarcinoma, in conjunction with inhibition of SIRT1, which was partially reversed by forced over-expression of the sirtuin [169]. Importantly, a similar inhibition of cell proliferation was not observed in nontransformed cells, indicating that the deregulation of the circadian machinery in the cancer cell is decisive. In accordance with these findings in prostate cancer, melatonin was found to repress, in breast cancer cells, *ROR*α, an effect that causes down-regulation of BMAL1 and SIRT1 [170]. In these cells, the aspect of circadian deregulation was particularly evident, because *Per2*, a core oscillator gene with tumor suppressor function, was not expressed.

The potential of melatonin for epigenetically modifying properties of tumors was also demonstrated on a larger scale in two studies using breast cancer cell lines. In MCF-7 cells treated with 100 nM melatonin, 22 miRNAs were differentially expressed compared to controls; 12 of them were up-regulated and 10 down-regulated [171]. At the near-physiological concentration of 1 nM, four up-regulated and one down-regulated miRNAs were detected. It would be of interest to further clarify whether the concentration dependence is indicative of an effective therapeutic dose. In two cases, increased miRNAs could be related to down-regulations of targeted mRNAs. In another case in which melatonin suppressed the expression of a miRNA, a corresponding rise in the targeted mRNA was observed [171]. Changes in DNA methylation patterns studied on a genome-wide scale in MCF-7 cells demonstrated a remarkably broad impact of melatonin on epigenetic modulation of gene expression [172]. At 1 or 100 nM melatonin, 8508 and 9196 methylated promoters, as well as 5256 and 6543 methylated CpG islands were detected, respectively. Among these, 2200 and 2824 genes carried methylations in both promoters and intragenic CpG islands. Compared to controls, 1605 and 3250 genes had hypermethylated, and 1925 and 1786 hypomethylated promoters, at 1 or 100 nM melatonin, respectively. In miRNA promoters, 15 and 20 were found to be hypermethylated, 4 and 9 hypomethylated. Numerous genes are listed, which are down-regulated by hypermethylation or up-regulated by hypomethylation at 1 nM melatonin, many of which are important signaling molecules or cancer-related [172]. The meaning of these findings is worth further specified analyses, but, regardless of such details, the two studies impressively show that melatonin is a broad-scale epigenetic modulator of gene expression.

## 9. The Central Nervous System, Neurogenesis, Neuropsychiatric Disorders and Melatonin

Recent years have shown that epigenetics is of utmost importance to the central nervous system, under aspects as different as neurodevelopment, neuroplasticity, learning and cognition, stress, neurotoxicology, addiction, psychopathology, and aging [173,174,175,176,177]. With regard to the complexity of the CNS and the extremely broad spectrum of modulatory actions, many fundamental questions remain to be answered. Consequently, the epigenetic effects described for melatonin hit various important aspects, but are not necessarily imbedded in a larger context of understanding.

Several findings collectively indicate that melatonin is involved in the maintenance of epigenomic traits that are typical for a healthy psychiatric state. Stress is known to induce epigenetic changes in the brain, with consequences to the development of neuropsychiatric disorders [155,156]. Melatonin antagonizes stress-induced behavioral changes [178] as well as a certain spectrum of neuropsychiatric symptoms [179,180,181]. Moreover, its levels are decreased under stress and various neuropathological conditions [181]. The possibility that melatonin counteracts epigenetic alterations in response to stress may be supported by a study on actions of agomelatine, a melatonergic agonist and 5-HT_2C_ serotonergic antagonist. This drug did not only reduce anxiety-like behavior in rats previously subjected to prenatal stress, but also corrected hippocampal levels of pCREB and mGlu2/3 and mGlu5 metabotropic glutamate receptors [182]. The persistence of the cellular changes after birth and its comparably easy reversal, speak for a likely epigenetic basis, although the precise mechanisms have not been studied. Moreover, the question remains as to what extent these actions are melatonergic, attributable to the serotonergic antagonism, or to an interplay between melatonergic and anti-serotonergic actions.

Another publication [183] has indicated a rather unfavorable epigenetic effect of melatonin, which has, however, remained purely hypothetical. The fact that the area postrema contains a relatively high density of melatonergic receptors has been taken as a clue for assuming that melatonin may be responsible for the acquisition of essential hypertension, by epigenetically shifting the set-point for a higher operating pressure via sympathetic activation. However, this hypothesis is not sufficiently based on mechanisms of melatonergic signaling and epigenetic causality. The same reservation may be necessary with another hypothesis dating back prenatal melatonin-induced epigenetic changes to the stage of oocytes [184], although this possibility should not be generally ruled out with regard to the demonstrable epigenetic potential of melatonin.

Direct epigenetic actions of melatonin in the nervous system have been documented. Niles and colleagues demonstrated area-specific changes in histone modification in response to melatonin, such as increased acetylation of histones H3 and H4 in the hippocampus, and of H4 in the striatum, but no such changes in midbrain and cerebellum [185]. A significant increase in histone H3 acetylation was also observed in the neural stem cell line C17.2, at melatonin concentrations of 0.1 and 1 nM [186]. In addition, rises in mRNA expression of HDAC3, HDAC5 and HDAC7 were described, which remained relatively moderate and were interpreted as a compensatory feedback to melatonin-induced hyperacetylation. In conjunction with these findings, up-regulations of the neural stem cell marker, nestin, and of the early neuronal marker, β-III-tubulin, were documented at the mRNA level, indicating, along with other criteria, the potential of melatonin as a differentiation factor in neurogenesis. The possible role of melatonin in stem cells has been also discussed in a review paper dealing with age-related impairments of self-renewal, factors involved such as Sox2 and possible epigenetic changes [187]. However, this specific area of stem cell conservation would require in the future more direct evidence for a role of melatonin. Several actions, summarized there, are also mentioned, in the present article, in Section 6, Section 7 and Section 9.

In the context of aging and age-related diseases, such as Alzheimer’s disease (AD), other epigenetic aspects have been addressed [10,188,189], with possible relevance to melatonin’s anti-aging effects [101], yet without consideration or in-depth discussion of a contribution by the indoleamine. AD-like memory deficits were investigated in a rat model using scopolamine toxicity [190]. The toxin caused decreases in EPAC1, EPAC2 (cAMP-regulated guanine nucleotide exchange factor-1, -2) and RagA (Ras-related GTP-binding protein), changes that led to an increase in *miR-124* and a decline of its target, *Egr1* mRNA (early growth response protein 1, alias NGFI-A, nerve growth factor-induced protein A). Melatonin reversed all these changes and attenuated memory and synaptic deficits [190].

Another emerging field of future relevance to melatonin concerns a broad spectrum of epigenetic alterations in neuropsychiatric disorders [14,191,192,193,194,195,196,197,198]. However, in these recent developments, the connection to melatonin is only occasionally addressed, or if so, poorly founded in mechanistic terms. To date, results from this area may only provide hints indirectly related to the involvement of circadian rhythms or to elsewhere published findings on ameliorations of symptoms by melatonin.

## 10. Conclusions

Although the existence of epigenetics has been known for decades, the exciting and largely unforeseen findings of recent years have substantially changed our understanding of gene expression. Many details remain to be clarified, but we now know that epigenetics is not only a matter of DNA methylation and histone modification, but in addition we have to consider the actions of previously unknown RNA species derived from a transcriptome that is by far larger than the sum of the transcripts from coding genes. We have also learned that mRNA stability is modulated in multiple ways, by miRNAs, lncRNAs and deadenylases. We are beginning to perceive that a reprogramming of methylated CpG islands is possible via hydroxylation of the methyl residues. In total, it seems hardly possible that the manifold ways of fine-tuning gene expression are not influenced by an extremely pleiotropic regulator molecule such as melatonin.

To date, several studies summarized in this review have directly demonstrated epigenetic effects of melatonin. However, many of these either describe rather global effects or have been obtained under very specific conditions present in tumor cells. Therefore, the body of evidence for melatonin’s role as an epigenetic regulator largely remains in the state of a proof of principle. What is needed in the future are detailed studies on specific genes of key importance under physiological conditions. Without any doubt, studies on pathological changes are also necessary, but the physiological basis has first to be known, before convincing conclusions can be drawn.

**Figure 2:**
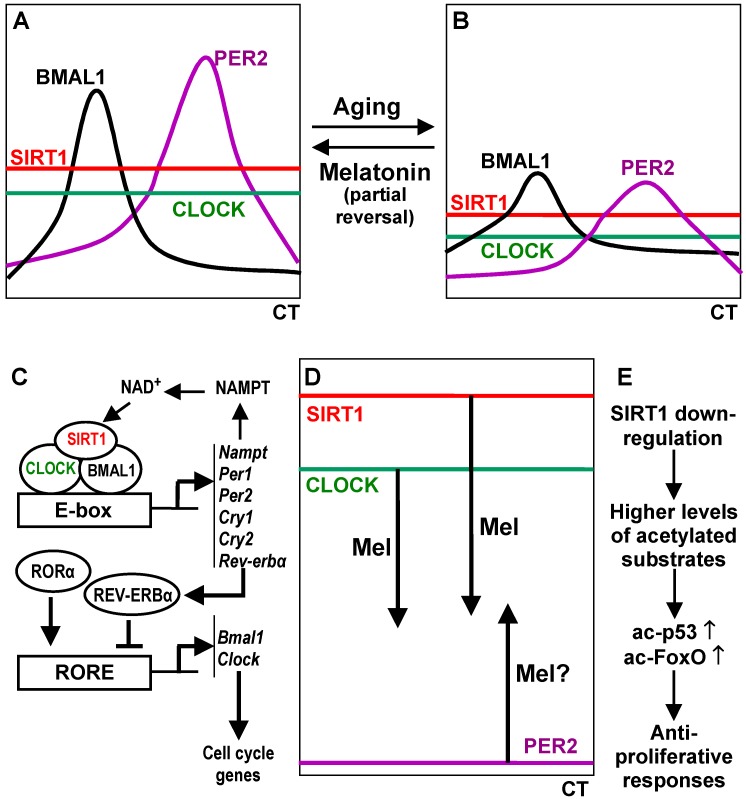
Simplified scheme of the role of the deacetylase SIRT1 in well-operating, in aged and by malignant transformation deregulated peripheral circadian oscillators (type: rodents). In order to reduce complexity, only a few selected clock components are represented. (**A**) Normal oscillations of BMAL1 and PER2 in young animals, in the absence of rhythmic CLOCK and SIRT1 expression levels (not to be confused with transcription-activating and enzyme activities, which underlie rhythmic regulation); (**B**) Aged oscillators exhibit reduced levels of all four proteins and flattened oscillatons; (**C**) Main interactions of the components discussed within the oscillator (*cf.* refs. [65,66,67,68,69]) and consequences to circadian cell-cycle gating; (**D**) Changes in deregulated oscillators of tumor cells that over-express SIRT1 and CLOCK (this is certainly not valid for all tumors). The tumor suppressor PER2 is down-regulated by promoter methylation and enhanced degradation because of deacetylation. A return of PER2 to cyclicity and to circadian gating of the cell cycle by melatonin has not been demonstrated, although proliferation is reduced; (**E**) Other consequences of SIRT1 down-regulation (*cf.* refs [167,168,169]. Abbreviations: CT: circadian time; Mel: melatonin; RORE: ROR response element.

In this context, the necessity of discriminating between dynamic and enduring changes induced by melatonin becomes particularly obvious (Figure 2). Melatonin, at least to a considerable degree, is part of the circadian oscillator system in a broader sense. As far as melatonin influences, under physiological conditions, the expression of oscillating genes, whether core or accessory genes, the initially induced changes can be expected to be reversed within the circadian cycle, although after-effects such as phase shifts or altered amplitudes may transiently persist. In such a system, the transitory nature of an effect will make it difficult or impossible to state that melatonin just up- or down-regulates a certain gene. For instance, a primary up-regulation may be followed by a more profound down-regulation in a later circadian phase, because of an increased amplitude. This should be considered for both core oscillator components such as *Per2* or *Clock* and accessory components such as *Sirt1* and *Ppar*γ. However, if effects of melatonin are studied in a poorly or even non-oscillating system such as cancer cells, which may have repressed some oscillator genes with tumor suppressor function, such as *Per2*, the situation is entirely different. In this case, a down-regulation of, e.g., *Bmal1* or *Sirt1* (*cf.* refs. [169,170]), may appropriately describe the finding, but this cannot be simply translated to nontumor cells. Some of the discrepancies between results obtained in tumor cells and in models of aging may be explained in this way. Nevertheless, effects of melatonin in cancer cells with deregulated circadian oscillators may be of immense value to combat this disease.

The body of knowledge concerning epigenetic changes in the circadian systems of organisms is already remarkably broad (*cf.* Table 1) and exceeds by far that of direct actions of melatonin. At first glance, one might be inclined to believe that the actions of melatonin as exerted in the SCN would not be of substantial importance, since they appear to be mainly restricted to minor phase shifts under normal physiological conditions. In fact, a circadian pacemaker can exist and operate in the absence of melatonin, as becomes obvious by all the numerous melatonin-deficient mouse strains.

However, this situation may be fundamentally different in peripheral oscillators (*cf.* refs. [37,38]). Although corresponding data have not been published on a broad scale, the oscillator in the mouse adrenal cortex provides a good example. In a melatonin-proficient strain, high amplitude cycles were observed in PER1, CRY2 and BMAL1, whereas no robust rhythmicity was apparent in a melatonin-deficient strain [75]. At least, in this case, the requirement of melatonin for a normal functioning of the peripheral oscillator is obvious. In the future, the elucidation of connections between melatonin and oscillators in epigenetic gene regulation may become an exciting field. This may be also of importance to conditions and pathologies that are associated with reductions of melatonin and/or chronodisruption.
